# On “Coherent control in the extreme ultraviolet and attosecond regime by synchrotron radiation” by Hikosaka et al, *Nat. Comm*. 10, 4988 (2019)

**DOI:** 10.1038/s41467-021-24024-9

**Published:** 2021-06-18

**Authors:** Kevin C. Prince, Bruno Diviacco

**Affiliations:** 1 Elettra - Sincrotrone Trieste S.C.p.A, Basovizza, Trieste Italy; 2 Centre for Translational Atomaterials, Swinburne University of Technology, Melbourne, VIC Australia

**Keywords:** Atom optics, Optical spectroscopy

**Arising from** Hikosaka et al. *Nature Communications* 10.1038/s41467-019-12978-w (2019)

Recently Hikosaka et al.^1,2^ reported the use of a pair of phased undulators at a synchrotron to achieve coherent control. They measured the fluorescent yield of He Rydberg states and found that the yield oscillated as a function of the relative phase of the undulators, to give what appeared to be Ramsey fringes. We have performed resonant photoemission experiments from solid samples using a similar undulator pair, and found the signal was proportional to the monochromatic flux of synchrotron light, which varied periodically with the phase. Rather than coherent control, we assign the intensity oscillations of Hikosaka et al to variations of the flux at the wavelengths of interest.

The coherence of electromagnetic radiation is immensely importance, and all light sources possess a degree of coherence described e.g. by the van Cittert–Zernike theorem.^3^ The partial coherence of synchrotron light has been exploited in some experiments, e.g. Ref. ^4^, but usually the coherence is increased by spatial filtering and frequency filtering with a monochromator. The coherent control described by Hikosaka et al assumes that the light consists of pairs of coherent optical wave packets, analogous to the Tannor–Rice scheme^5^. In this method, temporally separated, phase-coherent pulses excite a target and the time and phase between them is the control parameter, and gives rise to Ramsey fringes^6,7^.

In the experiment of Hikosaka et al.^1,2^, unmonochromated light excited Rydberg states of He and their populations were measured by detecting the fluorescence emitted when the Rydberg states decayed. The authors normalised this signal to the total flux emitted by the undulators, and found that the signal oscillated as a function of the phase between the undulators. However the population of the Rydberg state is not proportional to the total flux: it is proportional to the flux at the excitation wavelength.

In this work, we have performed a resonant excitation experiment on a solid sample. We measured the resonant photoemission intensity from a Re(0001) single crystal surface, using monochromatic synchrotron radiation, and a phased undulator pair. As well, we measured the monochromatic flux and the unmonochromated (total) flux as a function of the phase between the undulators. The spectra and absorption curve of the resonance are shown in Supplementary Fig. S1, and only the fluxes and spectral distributions are shown below. This experiment is conceptually similar to that of Hikosaka et al.^1,2^ because the He Rydberg state populations are determined by the monochromatic flux, not by the total flux.

Two major methods applied to study the interaction of light with matter may be described as monochromatic spectroscopy (our case), and broad band methods, such as conventional infra-red Fourier transform spectroscopy, where time delay is scanned. Hikosaka et al used the interference between the emission from two identical, phased undulators to modulate the intensity of the light, while we used a monochromator to select a narrow band of light. We detected a resonant photoemission signal, proportional to the population of ionic final states, while they detected fluorescence from He atoms excited to Rydberg states, which reflects the population of the corresponding final states. By selecting narrow resonances, their experiment was sensitive to the Fourier components of the light at the corresponding wavelength. Thus the experiments are not the same, but there is a correspondence between measurements of final state populations as a function of wavelength.

We measured signals at 38 eV as a function of the phase set between the two undulators, see Methods section. The total flux, the monochromatic flux and the photoemission intensity are shown in Fig. 1. The resonant signal is exactly proportional to the monochromatic flux, but is unrelated to the total flux, which varies only slightly with phase. If the total flux, which is nearly constant, is used for normalisation, the oscillation is retained, but if it is normalised to the monochromatic flux, the signal is constant. Supplementary Fig. S2 shows the measured and calculated monochromatic fluxes, which are in good agreement, indicating the reliability of our calculations.

**Fig. 1:**
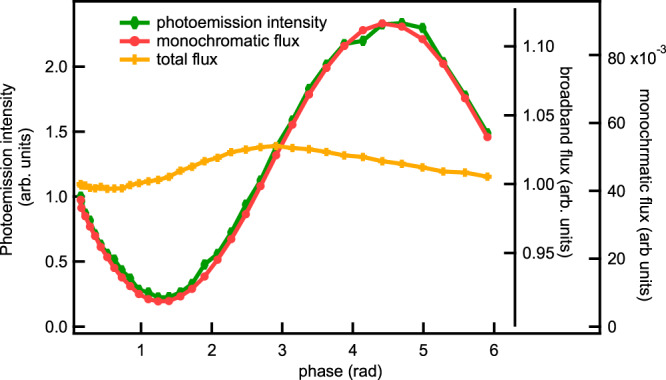
Measured resonant photoemission intensity at binding energy 2.5 eV (green curve), monochromatic flux (red curve) and total flux (orange curve) as a function of phase. The photoemission intensity and monochromatic flux are plotted on different axes from zero and scaled so the maxima overlap. Both curves vary from 10% to 100% of the maximum. Note the expanded scale for the total flux: the flux varies from 97% to 100% of the maximum. Photon energy: 38 eV.

The reason that the total flux varies very little with phase is clear from Fig. 2, showing the spectral distribution measured with a photodiode at fixed gap and variable phase. The total flux is the integral over photon energy of each curve. For undulators with phase = 0, a narrow peak centred at 38 eV is observed. In antiphase, destructive interference occurs for the central photon energy, but is constructive for nearby energies, compensating the loss of flux, leaving the total flux almost constant.

**Fig. 2:**
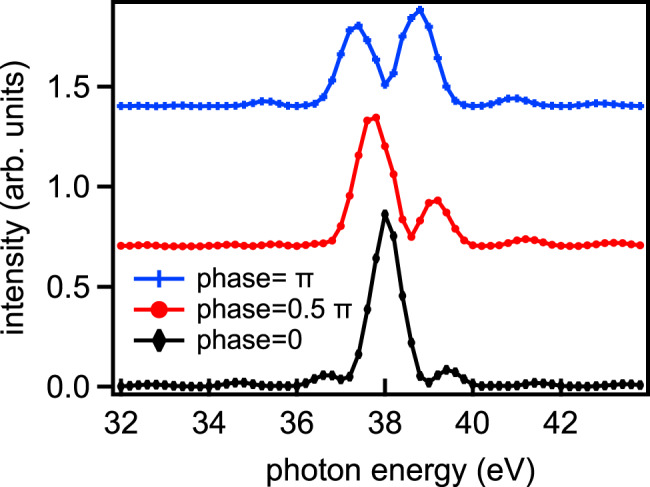
Measured spectral intensity as a function of photon energy with the undulator gaps optimised for 38 eV emission, and three values of the phase setting. Lower curve: phase = 0 rad; centre curve, phase = 0.5π rad; top curve, phase = π rad.

The present results indicate that the resonant signal from our sample does not depend directly on the phase setting of the light source, but is proportional to the monochromatic flux, which varies with the phase setting between the undulators. Hikosaka et al.^1,2^ did not use a monochromator, and the fluorescent signal from Rydberg states was normalised to the total flux of the insertion devices. As noted above, the fluorescent signal is determined by the intensity at the resonant wavelength, and because each resonance is very narrow, a correct normalisation requires a measurement of the monochromatic flux.

Hikosaka et al.^1,2^ presented a model of the process of generation of light from two phased undulators. They considered the light emitted as the result of an ensemble of coherent pulse pairs, in which the photons from each pair are distinguishable. However an ensemble of mutually incoherent short pulses, shifted in time, produces a long incoherent pulse. Because the two undulators are identical and have a fixed distance between them, their emission can interfere.

Rather than an ensemble of attosecond pulse emitters, a better optical analogue of the pair of undulators is an infra-red Fourier Transform spectrometer^8^, in which broadband radiation enters a Michelson interferometer. The beam is split into two replicas by mirrors; at the synchrotron, the replicas are created by two identical undulators. The phase is scanned in the FT spectrometer by moving mirrors, and in the undulators by delaying the electrons. In IR spectroscopy, the total signal is measured and Fourier transformed, whereas Hikosaka et al. measured signal at discrete wavelengths. Both a Michelson interferometer and phased undulators produce an outgoing beam in which constructive and destructive interference at various Fourier components occurs. FT spectrometry is not generally regarded as coherent control. In pump-dump coherent control^5^, temporally separated pulses are used, permitting for example a probe of the excited state population between the two pulses. This is impossible conceptually in the model of Hikosaka et al, because the 300 ps long pulse is an incoherent superposition of femtosecond pulses.

There are several definitions of coherent control^5–8^, e.g. “the ability to control the dynamics at various stages of a process as it evolves under the effect of a coherent source” ^9^, and indeed all definitions require a coherent source. Coherence be quantified by the first order coherence function g^(1)^, or the bandwidth-duration product. For a bandwidth of 10%, a central photon energy of 24 eV, and pulse duration of 300 ps, the product of frequency bandwidth and pulse duration is 1.8 × 10^8^, much higher than the Fourier transform limit of 0.441 for a Gaussian pulse. Thus the pulses used by Hikosaka et al., are far from being coherent in the sense of coherent control.

We conclude that the method of Hikosaka et al. is better described as interferometry but is not coherent control in the usual sense of the term. We suggest that the oscillations reported previously were due to optical interference in the source, and not to atomic coherence.

## Methods

The experiments were carried out at the Nanospectroscopy beamline^10,11^ at the Elettra synchrotron light source. The photon source consists of two identical Sasaki Apple II type undulator sections (each with 20 periods and period length 10 cm) and a phase shifter electromagnet between them^12–14^. This arrangement is similar to that of Hikosaka et al.^1,2^, who used a pair of undulators with 10 periods each. The energy of the electrons in the storage ring was 2.4 GeV, and the average electron bunch duration was ~60 ps. The light beam was spatially defined by an aperture of 0.5 mm (horizontal) by 0.9 mm (vertical) at a distance of 10 m from the source point in the second undulator.

The experimental station consists of a photoemission microscope^10^. The sample was the (0001) surface of a single crystal of Rhenium. The Re crystal was cleaned by repeated annealing cycles to about 1100 °C in molecular oxygen (P_O2_ = 1 × 10^−6^ mbar), followed by a high temperature flash (to about 2000 °C) under ultrahigh vacuum conditions. We measured the resonant photoemission of the valence band of Re. The resolving power (E/ΔE) of the monochromator was ~10^4^ for the photon energies considered. The photoelectron energy analyser of the photoemission microscope has an energy resolution of about 150 meV under typical operation conditions^10^. In the range from 35 to 45 eV photon energy, the 5d electrons of the valence band display resonant enhancement with a Fano line profile. The two paths leading to the interference are direct ionisation, and resonant excitation of the 4f_5/2,3/2_ electrons to the unoccupied states, followed by autoionization; both paths yield the same final state.

The total flux from the beamline as a function of phase was measured by setting the monochromator to zero order and inserting a photodiode into the beam after the exit slit, but before the experimental station. The monochromatic flux as a function of phase was also measured with the same photodiode. Identical phase scans were performed to measure the diode signal, and then to measure the resonant photoemission signal from the sample with the diode removed.

The theoretical data in Supplementary Fig. 2 were calculated using the program Spectra^13,15^.

## Supplementary information

Supplementary Information

## Data Availability

The data that support the findings of this study are available from the corresponding author upon reasonable request.
