# Supplementary material for: On “Coherent control in the extreme ultraviolet and attosecond regime by synchrotron radiation” by Hikosaka et al, Nat. Comm. 10, 4988 (2019)
Source: Nat Commun. 2021 Jun 18;12:3784. doi: 10.1038/s41467-021-24024-9 (PMC8213757; doi:10.1038/s41467-021-24024-9)
Supplement: Supplementary file 1 — Supplementary Information [file 41467_2021_24024_MOESM1_ESM.pdf]

Supplementary Information.

On "Coherent control in the extreme ultraviolet and attosecond regime by synchrotron radiation" by Hikosaka et al, *Nat. Comm.* **10**, 4988 (2019).

Kevin C. Prince and Bruno Diviacco.

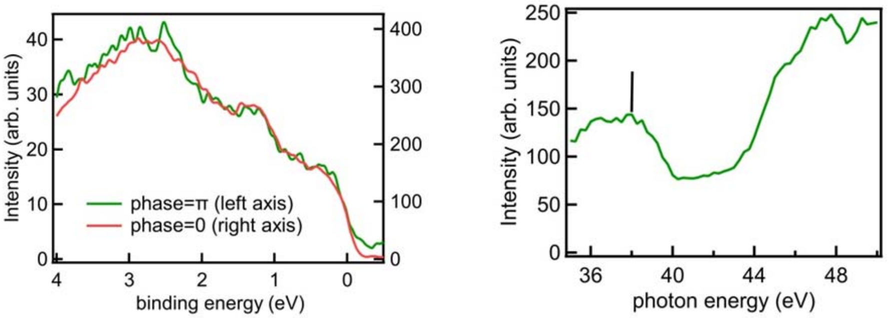

(a) (b)

Figure S1. Spectra of the sample. (a) Valence band spectrum of the sample with the undulators set in phase (phase=0 rad), and in antiphase (phase=π rad), photon energy 38 eV. (b) Constant initial state spectrum: intensity of the Re valence band at a binding energy of 2.5 eV, normalized to the photon flux measured by a gold mesh placed in the beam path, as a function of photon energy. The line marks the photon energy used for the following studies.

Figure S1(a) shows valence band spectra of the sample at 38 eV photon energy, with the undulator phases set to 0 and π. The spectra show the same features, except that the noise and background levels for the antiphase spectrum are higher because the signal level is lower, due to the reduced intensity. Figure S1(b) shows a constant initial state spectrum at a binding energy of 2.5 eV. There is a clear resonance, which occurs due to interference between two paths: direct photoemission of the Re 5d electrons; and 4f->5d transitions followed by Auger decay of the 4f hole with the emission of a 5d electron. We focus on these resonant states excited by 38 eV photons, at a binding energy of 2.5 eV.

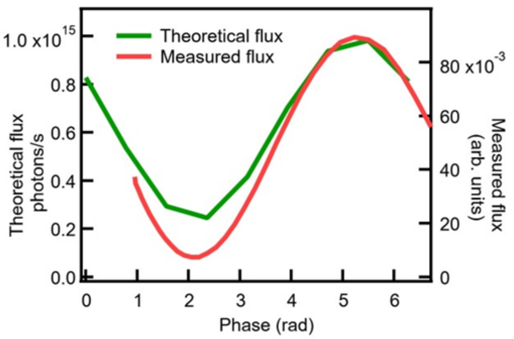

Figure S2. Calculated and measured monochromatic flux at 38 eV.

Figure S2 shows the calculated and measured flux as a function of phase, and they agree well.
